# Nature of Life

**Published:** 1871-06

**Authors:** 


### Nature of Life.

We clip the following from the Philadelphia *Sunday Dispatch* of the 25th ultimo. Its perusal cannot fail to provoke a "molecular" smile, and to sharpen the " protoplastic' appetite of our readers :

" Professor Poey, of Lycoming county, in this State, has been trying to tell us what 'life' is. According to Poey, 'Life results from a double molecular motion, general and continuous, of composition and of decomposition in relation to the organism and the inorganic medium. The medium is the combination of external agents, physical and chemical, proper to furnish to the organism the principles necessary for its nutrition and the manifestations of the properties ot the anatomical elements.'

Strange! how Error fastens itself in the human mind, and by its rank growth chokes the tender plant of Truth! During all the fourscore years of our existence we have cherished the fond delusion that Life was rather an immorigerous outgrowth of a retiary paradox, which engrafted upon the persiflage a mephetic diapason, causing it to permeate the neurosthenic rhomboid, and so producing isothermally protoplastic vitality. That is what *we* thought life was. But we see the mistake now, since Poey mentions it! "It is hard, though—very, very hard—to see the idols of our youth thus thrown down and broken one after the other. And by a man named Poey, too! It will make our whole Christmas season sad."—*Medical Times.—Can. Phar. Journal.*

